# Measles Tracker: a near-real-time data hub for measles surveillance

**DOI:** 10.1093/jamiaopen/ooaf062

**Published:** 2025-06-27

**Authors:** Francesco Branda, Maria Tomasso, Mohamed Mustaf Ahmed, Massimo Ciccozzi, Fabio Scarpa

**Affiliations:** Unit of Medical Statistics and Molecular Epidemiology, University Campus Bio-Medico of Rome, Rome, 00128 , Italy; Translational Health Research Center, Texas State University, San Marcos, TX 78666, United States; Department of Computer Science, Texas State University, San Marcos, TX 78666, United States; Faculty of Medicine and Health Sciences, SIMAD University, Mogadishu 252, Somalia; Unit of Medical Statistics and Molecular Epidemiology, University Campus Bio-Medico of Rome, Rome, 00128 , Italy; Department of Biomedical Sciences, University of Sassari, Sassari, 07100 , Italy

**Keywords:** measles, surveillance, real-time monitoring, infectious disease tracking, public health data

## Abstract

**Objectives:**

Measles continues to pose a serious threat to global public health, fueled by declining vaccination rates, international travel, and persistent immunization gaps. Early outbreak detection and response remain hampered by fragmented surveillance systems, which often lack interoperability and limit data accessibility.

**Materials and Methods:**

To address the major limitations of current measles surveillance systems—including data fragmentation and lack of standardization—we developed Measles Tracker, an integrated near-real-time data hub that centralizes and harmonizes measles surveillance data in the United States using publicly available sources. The system aggregates data from multiple layers, including: (1) official reports from public health agencies, (2) epidemiological surveillance bulletins, and (3) outbreak reports, mainly captured through news websites or via news aggregators. The platform architecture implements (1) geospatial normalization of key epidemiological variables (case counts, vaccination coverage, age-stratified incidence) and (2) dynamic visualization interfaces to support coordination of evidence-based response.

**Results:**

Measles Tracker enhances situational awareness by integrating disparate data streams in near real-time, enabling rapid geospatial detection of outbreak clusters, mapping vaccination gaps, and supporting dynamic risk stratification of vulnerable populations. It is intended exclusively as a complementary tool to official public health systems, providing educational and situational awareness without interfering with contact tracing, vaccination, or outbreak control activities.

**Conclusions:**

As a centralized, scalable tool, Measles Tracker advances measles surveillance by leveraging digital epidemiology principles. Future iterations will incorporate additional data streams (eg, climate variables, genomic surveillance) and advanced analytics (eg, machine learning for risk prediction, network models for transmission dynamics) to further optimize outbreak preparedness and resource allocation. This framework underscores the transformative potential of integrated data systems in global measles elimination efforts.

## Background

The response to infectious disease outbreaks has historically relied on the collection and analysis of disparate epidemiological data to guide public health interventions.^1^ During the COVID-19 pandemic, we saw a proliferation of initiatives to collect and share data on cases, deaths, vaccinations, testing rates, nondrug interventions, and genomic sequencing. Although these initiatives have provided valuable information, they have also highlighted the difficulties in integrating fragmented data streams into a coherent framework for timely analysis and effective decision making.^2^^,^^3^

For measles—a highly contagious but vaccine-preventable disease—the difficulties associated with data fragmentation and reporting delays remain particularly relevant.^4^^,^^5^ Understanding the historical evolution of measles surveillance in the United States offers valuable insights into how current systems developed—and what their critical issues remain today. Early surveillance efforts were based primarily on mortality statistics in the prevaccine era, but significant progress was made with the introduction of national elimination targets in the late 20th century. A pivotal moment occurred in 1978, with the launch of a coordinated national strategy to eliminate native measles through intensified vaccination campaigns and enhanced surveillance.^6^ This approach was further refined in the following decades, particularly with the introduction in the 1980s and 1990s of the second dose of measles, mumps, and rubella (MMR) vaccine as a response to outbreaks in school settings—signaling an important transition to increasingly data-driven health policies.^7^^,^^8^ These developments marked significant progress but also revealed persistent challenges. Early failures to meet elimination targets revealed gaps in vaccination coverage and limitations in surveillance-criticality infrastructure that still undermine measles control today.^9^

In recent years, such vulnerabilities have resurfaced. Measles has reemerged in several countries, including the United States, mainly due to declining vaccination coverage, increased vaccine hesitancy, and structural weaknesses in immunization programs.^10^ In 2019, the United States experienced the highest number of measles cases in 30 years, with outbreaks linked to international imports and subsequent transmission within communities with low vaccination coverage.^11^^,^^12^ These events have renewed attention to the urgency of more robust and responsive surveillance systems that can identify transmission patterns early, identify populations at risk, and enable rapid and targeted interventions by health authorities.^13^^,^^14^

The evolution of measles surveillance in the United States is summarized in Table S1, which outlines the major milestones from 1912 to the present. This process is overseen primarily by the National Notifiable Diseases Surveillance System (NNDSS), which serves as the basic infrastructure for monitoring notifiable diseases nationwide. Established in 1912, the NNDSS is a collaborative, multilevel system managed by the Centers for Disease Control and Prevention (CDC) in partnership with state and territorial health departments and the Council of State and Territorial Epidemiologists.^15^^,^^16^ Originally focused on a limited number of acute infectious diseases, the system has expanded substantially to include nearly 120 conditions ranging from infectious diseases like measles to noninfectious and emerging threats such as COVID-19 and *Candida auris*^17^^,^^18^ (see Table S2). The NNDSS operates as a “system of systems,” structured to collect, standardize, and disseminate disease surveillance data from clinicians, laboratories, hospitals, and local health departments to the national level.^19^ Health-care providers are legally required to report notifiable conditions to local authorities; those data are then transmitted—on a voluntary basis—to the CDC. This process supports timely outbreak detection, ongoing trend monitoring, evaluation of intervention effectiveness, and international health obligations such as those under the International Health Regulations. A comprehensive overview of the main platforms and reports that publish NNDSS data, including their scope and update frequency, is provided in Table S3.

Despite its critical role, the NNDSS is not without limitations. The voluntary nature of national notification and the variability in state-level reporting practices introduce inconsistencies and delays that may hinder rapid situational awareness during outbreaks. The completeness and accuracy of data are also influenced by diagnostic capacity, changes in case definitions, and jurisdictional priorities. These limitations have become especially apparent in the context of rapidly evolving public health threats like measles, where timeliness is essential.^20^ A clear example of the operational complexity involved in measles surveillance is illustrated by the detailed guidelines issued by state health departments. For instance, the Washington State Department of Health outlines standardized procedures covering disease reporting timelines, case definitions, diagnostic recommendations, laboratory services, and outbreak control measures.^21^ These guidelines reinforce the existence of well-defined practices at the state level but also highlight the challenge of harmonizing such procedures across jurisdictions to ensure consistency, timeliness, and interoperability at the national level.

To address these shortcomings, the CDC launched the NNDSS Modernization Initiative, which aims to improve data interoperability, streamline reporting via Electronic Case Reporting and Electronic Laboratory Reporting, and implement Health Level Seven messaging standards. While these advances represent critical steps forward, full implementation across all jurisdictions remains ongoing, and significant variation persists in data timeliness and completeness.

The CDC’s most recent reports point to a sharp increase in measles cases in 2024, attributed largely to international imports and a high prevalence of cases among unvaccinated individuals,^22^ further underscoring the need for complementary and agile tools to strengthen traditional surveillance infrastructure. Several studies have demonstrated the potential of these approaches: for example, analysis of online search behavior using Google Trends has proven effective in detecting early signs of measles outbreaks in countries such as Japan and Italy. In these cases, strong correlations were observed between search activity and official case data at the national and regional levels.^23^^,^^24^ As a digital complement to traditional systems, Measles Tracker is proposed as a transparent and open public platform that aggregates, harmonizes, and visualizes data for educational purposes and to improve situational awareness. It is important to clarify that Measles Tracker is not an official surveillance system and does not collect confidential or individual-level case data. The platform operates entirely on publicly available information and does not aim to alter, replace, or interfere with formal public health systems such as NNDSS, nor with critical outbreak response activities including case investigation, contact tracing, isolation, or vaccination campaigns conducted by CDC, state, or local health departments. All data used are anonymized and updated regularly based on public sources, ensuring full respect for existing reporting pipelines and outbreak control actions.

## Methods

**Figure 1.**
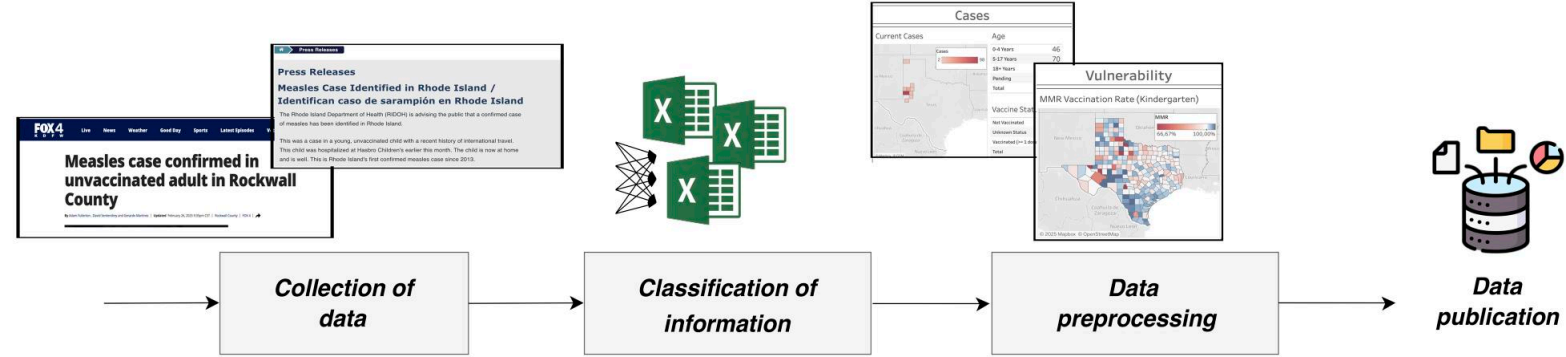
Schematic overview of the main steps from digitization to construction of the final dataset.

The methodology involved a systematic approach to data collection, integration, and processing, as summarized in Figure 1.

Given the current scale of data collection, the process is primarily manual, as the volume of data is still manageable. Data were collected from a wide range of sources, including local health departments such as the Texas Department of State Health Services (https://www.dshs.texas.gov/news-alerts/measles-outbreak-2025) and national public health agencies like the CDC (https://www.cdc.gov/measles/data-research/index.html). Moreover, to find additional details for each case, we augment these data with online reports, mainly captured through news websites (eg, https://www.cidrap.umn.edu/measles) or via news aggregators (eg, https://bnonews.com/).

Updates are usually made shortly after the new reports are released, which occur on Tuesdays and Fridays, ensuring near-real-time incorporation of emerging information into the dataset.

To address the heterogeneity in data formats, granularity, and terminology across sources, we implemented a 2-tiered approach to data harmonization. First, we defined a set of core variables that were standardized across all datasets to ensure consistency and interoperability. These variables included the geographic information (state, county, and city names standardized using Federal Information Processing System codes), temporal information (year and month of case reporting formatted as YYYY-MM), and case counts (number of reported measles cases with additional details on age groups, hospitalization rates, and vaccination coverage where available). For variables that could not be fully standardized due to source-specific variations, we included flexible fields such as the “details_cases” field, which allowed for free-text descriptions of specific cases or outbreaks, enabling researchers to include nuanced information that may not fit into predefined categories. A comprehensive data dictionary was created to define each variable (as summarized in Tables S4 and S5), its format and meaning, which serves as a reference for researchers and public health officials to ensure that the data are interpreted correctly.

Data processing and analysis were conducted using Python, leveraging libraries such as Pandas, NumPy, and SciPy for data manipulation, cleaning, and statistical analysis. These tools allowed us to perform tasks such as data aggregation, trend analysis, and visualization. For public-facing visualizations, Tableau was used to create interactive dashboards that display key metrics such as case counts, vaccination rates, and outbreak hotspots. All data used in the framework are aggregated to ensure privacy, and any individual-level data collected from sources are already anonymized by the originating entities.

The dataset is managed with Git, so researchers can monitor changes over time and revert to previous versions if necessary. To ensure data integrity, data quality checks are designed to identify inconsistencies, missing values, or outliers, ensuring that only high-quality data are included in the final dataset. Specifically, the system performs validation checks on data formats (eg, ensuring dates are in YYYY-MM-DD format), verifies the completeness of required fields (eg, case counts, geographic information), and flags anomalies such as unusually high or low values that may indicate reporting errors. Additionally, a dedicated bulletins folder has been established to archive all official documents released by government agencies, such as health departments and public health organizations. This folder serves as a reference source, allowing researchers to cross-check and verify data retrospectively. By maintaining a comprehensive record of official bulletins, the Measles Tracker ensures transparency and traceability, enabling users to validate data accuracy and resolve discrepancies effectively.

## Use cases

The Measles Tracker supports a wide range of use cases, including spatiotemporal analysis to identify hotspots and trends over time, vaccination coverage monitoring to track rates at the state, and county levels, predictive modeling to forecast future outbreaks based on historical data, and public health dashboards to provide actionable insights to policymakers and the general public. To illustrate the value of these data, we show 2 simple analyses that researchers could do using these data, with different levels of effort.

### Example 1. The biogeography of disease outbreaks

**Figure 2.**
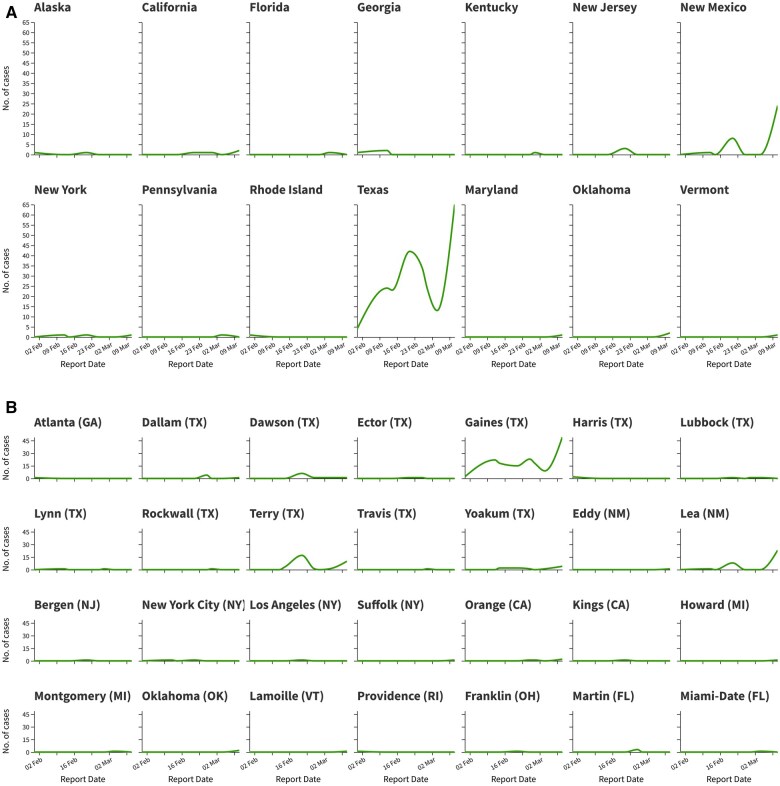
The distribution of outbreaks through time. (A) Number of cases per day and state. (B) Number of cases per day and county.

Figure 2 illustrates the temporal and geographic distribution of measles outbreaks in the United States, highlighting key trends and critical areas. Figure 2A reveals that Texas is the epicenter of the epidemic, with a significant concentration of cases reported across multiple counties. For instance, Gaines County in Texas experienced a sharp increase in cases, with 22 confirmed cases on February 12, 2025, and a peak of 23 cases on February 26, 2025. Similarly, Terry County reported a notable surge, with 17 cases on February 21, 2025. Other states, such as California and New York, also reported sporadic outbreaks, though on a smaller scale. For example, New York City recorded 1 case on February 12 and February 21, 2025, while California reported 1 case on February 21 and February 26, 2025. At the county level (Figure 2B), the data highlight specific hotspots within states. In addition to Gaines and Terry Counties in Texas, Dawson County reported 6 cases on February 21, 2025, and Lea County in New Mexico experienced a significant outbreak, with 8 cases on the same date. These localized outbreaks underscore the importance of granular data in identifying high-risk areas and implementing targeted interventions. The temporal analysis indicates a gradual escalation in the spread of measles, with a major peak observed on February 21, 2025, particularly in Texas. This trend aligns with the broader pattern of measles resurgence in the United States, driven by declining vaccination rates and vaccine hesitancy in certain communities. The data also reveal that while some regions, such as Florida and Pennsylvania, reported isolated cases later in the timeline (eg., 1 case in Florida on March 4, 2025, and 1 case in Pennsylvania on the same date), the overall burden of the disease remains concentrated in Texas and a few other states.

### Example 2. Near-real-time public health dashboards for outbreak monitoring

**Figure 3.**
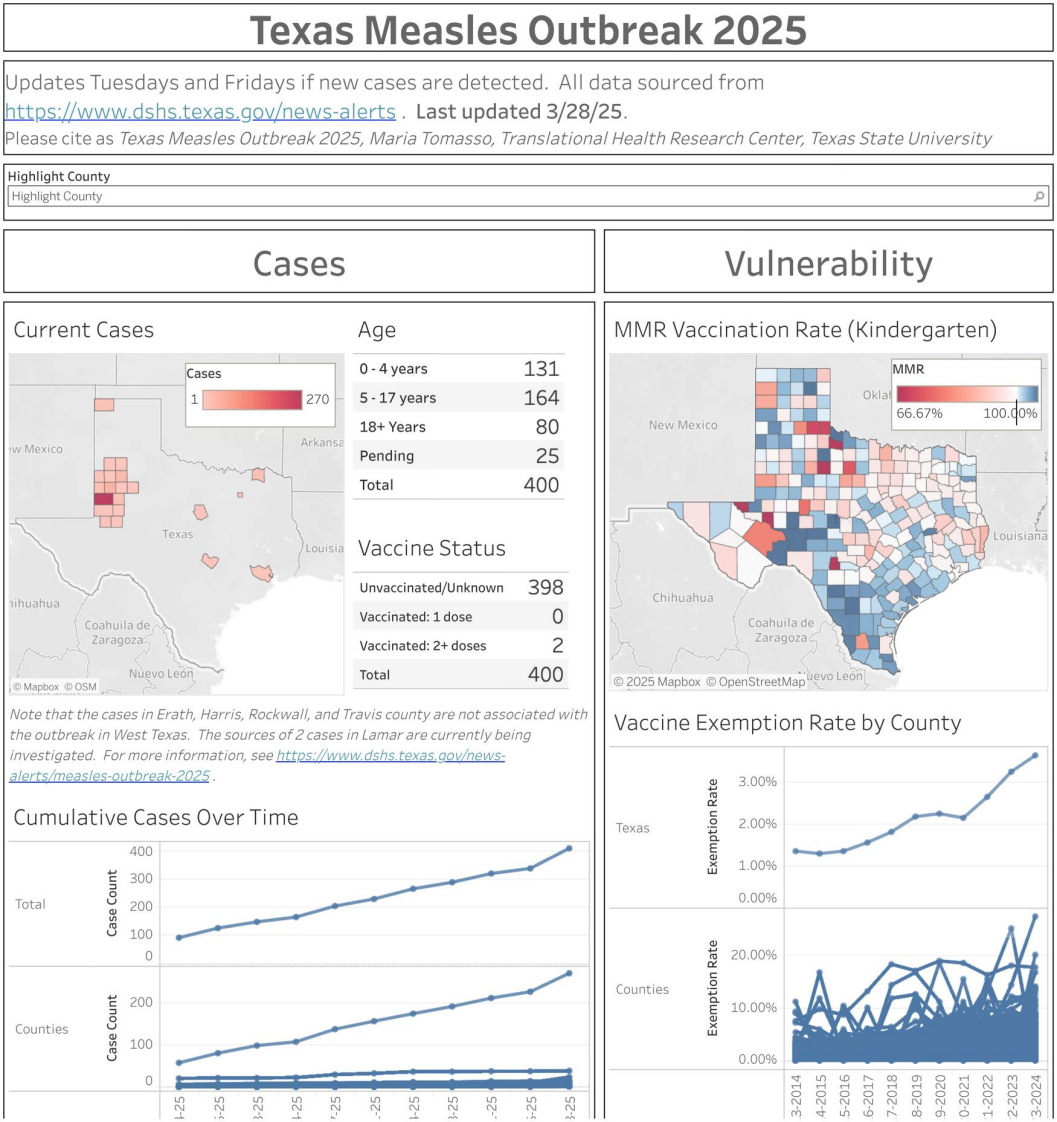
Texas measles outbreak 2025 dashboard.

Figure 3 illustrates a public dashboard on the 2025 measles epidemic in Texas, available at https://public.tableau.com/app/profile/maria.tomasso/viz/measles_2025/Dashboard1. The left-hand column of the dashboard features visualizations showing case counts at both the state and county level, along with detailed tables that break down current cases by age group and vaccination status. The right-hand column provides county-level metrics on vulnerability to measles outbreaks, including MMR vaccination rates among kindergarten children and conscience exemption rates for vaccines.

## Discussion

The persistent fragmentation of epidemiological data continues to be a major obstacle to the effectiveness of infectious disease surveillance and the timely implementation of public health interventions. For diseases such as measles, which require rapid response to prevent transmission, delays in the availability or integration of data on cases, vaccination coverage, and transmission dynamics can significantly undermine the ability to respond and contain outbreaks. This critical issue is exacerbated by the fact that crucial information is often dispersed among disparate and uncoordinated sources—including local and state health departments, clinical facilities, laboratories, and international agencies—each with its own standards, timing, and reporting formats.

The Measles Tracker was developed as a flexible and transparent tool with the goal of improving visibility and responsiveness through the aggregation of publicly available data in near real-time. Although it does not function as an automated or truly real-time system, it provides frequent updates—usually within 24-72 h of publication of sources—consolidating heterogeneous information streams such as official bulletins, case reports, and journalistic sources. This aggregation enables timely identification of case clusters and vaccine gaps, helping to strengthen situational awareness in public health. A major strength of the platform lies in its adaptability: the open architecture allows for future integration of additional sources, such as electronic health records, school absenteeism data, wastewater monitoring, or even genomic data. By diversifying input data, the system could make it easier to understand drivers of prevalence, such as immune or social inequalities.

Innovation in surveillance must also be based on a sound understanding of the historical context. The path of measles control in the United States—from the collection of mortality data to the introduction of national elimination goals to the adoption of double-dose vaccination—offers important lessons about the evolution of data-driven strategies. Key policies such as the 1978 elimination goal and the reforms of the 1980s and 1990s show how surveillance can effectively guide health decisions. At the same time, initial failures to achieve these goals revealed structural vulnerabilities: inadequate vaccination coverage, uneven data quality, and undersized local infrastructure. These lessons remain highly relevant, especially in light of the reemergence of measles in settings characterized by vaccination decline, vaccination hesitancy, and health inequalities.

Tools such as the Measles Tracker should not be understood as isolated technical solutions, but as elements of a broader health ecosystem that requires coordination, transparency, and ongoing adaptability. Similar digital approaches have already proven their effectiveness in other contexts, such as in tracking H5N1 avian influenza outbreaks using real-time epidemiological data, contributing to public health preparedness and awareness.^25^ These experiences reinforce a central principle: health surveillance, while remaining anchored in traditional infrastructures, increasingly benefits from agile and complementary systems that can synthesize diverse information into actionable knowledge.

In an era marked by growing global health threats and increasing data complexity, the integration of real-time or near-real-time surveillance tools constitutes a strategic investment in outbreak preparedness and response. The Measles Tracker, through the use of open data, dynamic visualizations, and a modular design, contributes to this vision. Its value lies not in replacing official systems, but in its ability to promote transparency and provide all stakeholders with timely, interpretable, and relevant information.

## Supplementary Material

ooaf062_Supplementary_Data

## Data Availability

The data presented in this study are available at https://tinyurl.com/USA-measles.
