# Supplementary material for: Measles Tracker: a near-real-time data hub for measles surveillance
Source: JAMIA Open. 2025 Jun 27;8(3):ooaf062. doi: 10.1093/jamiaopen/ooaf062 (PMC12203508; doi:10.1093/jamiaopen/ooaf062)
Supplement: ooaf062_Supplementary_Data [file ooaf062_supplementary_data.docx]

**Measles tracker: A near-real-time data hub for measles surveillance**

Authors: **Francesco Branda^1,*^PhD**, **Maria Tomasso^2,3^ MS (PhD candidate)**, **Mohamed Mustaf Ahmed^4^ MD**, **Massimo Ciccozzi**^1^ **PhD**, **Fabio Scarpa^5^ PhD**

Affiliations

1. Unit of Medical Statistics and Molecular Epidemiology, University Campus Bio-Medico of Rome, Rome, Italy
2. Translational Health Research Center, Texas State University, San Marcos, USA
3. Department of Computer Science, Texas State University, San Marcos, USA
4. Faculty of Medicine and Health Sciences, SIMAD University, Mogadishu, Somalia
5. Department of Biomedical Sciences, University of Sassari, Sassari, Italy

Corresponding author: Mohamed Mustaf Ahmed, Faculty of Medicine and Health Sciences, SIMAD University, Mogadishu, Somalia (momustafahmed@simad.edu.so). ORCID: https://orcid.org/0009-0006-5991-4052

**Supplemental Material Content**

**Table S1.** Recent changes to the National Notifiable Disease List (2024-2025).

**Table S2.** Key data platforms and publications for NNDSS data.

**Table S1.** Key milestones in the evolution of measles surveillance in the United States, highlighting historical shifts in surveillance strategies, vaccination policies, and ongoing challenges to timely outbreak detection and reporting.

| **Period** | **Milestone** | **Key events and strategies** |
| --- | --- | --- |
| 1912 | Measles became a nationally notifiable disease in the United States. | State and territorial health authorities, in conjunction with the Public Health Service, recommended monthly reporting of 10 infectious diseases, including measles. |
| 1912-1920s | All states participated in the reporting of measles cases to the US Public Health Service. | By the mid-1920s, all states were participating in measles case reporting. |
| Pre-1963 | An average of 549,000 measles cases and 495 measles deaths were reported annually, with an estimated 3-4 million cases occurring each year. | Early surveillance data documented the significant health burden of measles. |
| 1963 | Measles vaccine was licensed in the United States. | The advent of the measles vaccine era began. |
| 1963-1968 | Reported measles cases decreased by 95% due to vaccination programs. | Measles surveillance demonstrated the significant impact of vaccination programs. |
| 1978 | The CDC set a goal to eliminate measles from the United States by 1982. | A national strategy aimed to eliminate indigenous measles through aggressive immunization and surveillance. |
| 1979 | A standard clinical case definition for measles was adopted. | A standard clinical case definition was adopted to permit more uniformity in reporting clinically confirmed cases. |
| 1989 | A second dose of the MMR vaccine was recommended for all children. | Introduction of a routine second dose of the MMR vaccine in response to school-based outbreaks. |
| \| 1989–1991 \| \| --- \|  \|  \| \| --- \| | A resurgence of measles occurred with over 55,000 cases and 123 deaths reported. | The resurgence highlighted gaps in immunization coverage and the need for more robust surveillance strategies. |
| 2000 | Measles was declared eliminated in the United States (absence of continuous disease transmission for greater than 12 months). | This achievement was attributed to a highly effective vaccination program and better measles control in the Americas region. |
| 2001-2019 | 3,873 measles cases were reported in the United States, with a median of 86 cases per year. Most cases were among unvaccinated individuals. | During this period, the objectives of measles surveillance were to detect cases and small transmission chains, detect imported cases, and document progress toward elimination. |
| 2019 | The United States experienced the highest number of measles cases (1,274) since 1992. | This resurgence threatened the US's measles elimination status. |
| 2024 | Sharp increase in measles cases attributed to international imports and unvaccinated individuals. | The US verified the ongoing elimination of measles. |
| Current (2025) | Over 1,000 measles cases reported across the US, with a significant outbreak in Texas. | As of March 13, 2025, 301 confirmed cases were reported across 15 jurisdictions. |

**Table S2.** Recent changes to the National Notifiable Disease List (2024-2025).

| **Year of Change** | **Disease/Condition** | **Action** | **Reason for Change** |
| --- | --- | --- | --- |
| 2025 | COVID-19 | Removed from Nationally Notifiable List | Transition of public health response to routine surveillance methods |
| 2025 | Rift Valley fever virus | Added to Nationally Notifiable List | New emerging infectious disease requiring national monitoring and response |
| 2025 | Anthrax | Reporting Change | Alignment with updated CSTE position statement to include all anthrax toxin-producing *Bacillus* species |
| 2025 | Babesiosis | Reporting Change | No longer included in weekly tables but remains in annual tables |
| 2025 | Leprosy (Hansen's disease) | Reporting Change | Alignment with updated CSTE position statement to publish both confirmed and probable cases |
| 2025 | Novel influenza A | Reporting Change | Alignment with updated CSTE position statement to include both confirmed and probable cases |
| 2025 | Viral hemorrhagic fevers | Reporting Change | Alignment with updated CSTE position statement to publish only confirmed cases |
| 2025 | Rubella | Reporting Change | Alignment with updated CSTE position statement to publish only confirmed cases |
| 2025 | Chagas Disease | Added as Condition Under Standardized Surveillance | New condition under standardized surveillance (not in weekly tables) |
| 2025 | Firearm-related injury | Added as Condition Under Standardized Surveillance | New condition under standardized surveillance (not in weekly tables) |
| 2024 | Invasive *Cronobacter* infection among infants | Added to Nationally Notifiable List | New condition requiring national monitoring and coordinated response |
| 2024 | Zika virus non-congenital and congenital infection | Removed from Nationally Notifiable List | Shift in public health threat posed by the virus |
| 2024 | Ehrlichiosis and anaplasmosis | Reporting Change | Removed from weekly tables but remain in annual tables; undetermined ehrlichiosis/anaplasmosis no longer nationally notifiable |
| 2024 | Hepatitis B and C | Reporting Change | Inclusion of confirmed and probable cases in weekly tables |
| 2024 | Toxoplasmosis | Added as Condition Under Standardized Surveillance | New condition under standardized surveillance (not in weekly tables) |
| 2024 | Congenital cytomegalovirus infections | Added as Condition Under Standardized Surveillance | New condition under standardized surveillance (not in weekly tables) |

**Table S3.** Key data platforms and publications for NNDSS data.

| **Platform/Publication Name** | **Type** | **Host/Publisher** | **Key Features/Data Provided** | **URL (where applicable)** |
| --- | --- | --- | --- | --- |
| Morbidity and Mortality Weekly Report (MMWR) | Weekly Report | CDC | Provisional data and summaries of notifiable diseases | <https://www.cdc.gov/mmwr/index.html> |
| Summary of Notifiable Diseases, United States | Annual Summary | CDC | Finalized annual data on notifiable disease occurrence | <https://www.cdc.gov/mmwr/mmwr_nd/index.html> |
| CDC WONDER | Interactive Database & Visualization Tool | CDC | Weekly and annual NNDSS data, interactive queries, data visualization | <https://wonder.cdc.gov/> |
| data.cdc.gov | Public Data Portal | CDC | Weekly NNDSS data and other public health datasets | <https://data.cdc.gov/> |
| CDC Stacks | Data Repository | CDC | Weekly and annual NNDSS tables in PDF and text formats | <https://stacks.cdc.gov/> |
| NNDSS Data Visualization Tool (Australia) | Interactive Visualization Tool | Australian Government Department of Health and Aged Care | Interactive graphs and tables for notifiable diseases in Australia | <https://www.health.gov.au/our-work/nndss> |

**Table S4.** Data dictionary for near-real-time collection.

| **Variables** | **Definition** | **Format** |
| --- | --- | --- |
| source | \| The origin or provider of the data (e.g., health department, hospital, etc.) \| \| --- \| | String |
| year | Year of the reported case(s) | YYYY |
| month | Month of the reported case(s) | MM |
| state_name | Full name of the State in which the case resides | String |
| state_code | \| Numeric code representing the state-level Federal Information Processing System (FIPS) code \| \| --- \| | Numeric |
| county_name | Full name of the County in which the case resides | String |
| county_code | \| Numeric code representing the County (i.e., FIPS code) \| \| --- \| | Numeric |
| city_name | Full name of the City in which the case resides | String |
| cases_count | Number of reported measles cases | Numeric |
| details_cases | Additional information or notes about the case(s) | String |
| age_group | Age category of the affected individuals (i.e., <5, 5-19, >20, Unknown) | String |
| case_count_per_age | Number of cases within the specified age group | Numeric |
| percentage_case_count_per_age | Percentage of total cases represented by the specified age group | Numeric |
| hospitalized_cases_per_age | Number of cases within the age group that required hospitalization | Numeric |
| percentage_hospitalized_count_per_age | Percentage of hospitalized cases within the specified age group | Numeric |
| last_update | Date when the data was last updated | YYYY-MM-DD |

**Table S5.** Data dictionary for historical cases.

| **Variables** | **Definition** | **Format** |
| --- | --- | --- |
| source | \|  \| \| --- \|   The origin or provider of the data (e.g., health department, hospital, etc.) | String |
| geography | \| Geographic location (e.g., state) \|  \| \| --- \| --- \| | String |
| week_start | Start date of the epidemiological week | YYYY-MM-DD |
| week_end | End date of the epidemiological week | YYYY-MM-DD |
| school_year | \| Academic year for which the Measles, Mumps, and Rubella (MMR) coverage data is reported (e.g., 2022-2023) \| \| --- \| | YYYY |
| population_size | Total population size for the geographic area | Numeric |
| percent_surveyed | Percentage of the population surveyed for MMR coverage | Numeric |
| survey_type | Type of survey conducted (e.g., school-based, community-based) | String |
| categories | Categories or subgroups for coverage estimates (e.g., 90-94.9%) | String |
| cases | Number of measles cases reported during the week | Numeric |
